# The Scoring Challenge of Emotional Intelligence Ability Tests: A Confirmatory Factor Analysis Approach to Model Substantive and Method Effects Using Raw Item Scores

**DOI:** 10.3389/fpsyg.2022.812525

**Published:** 2022-03-10

**Authors:** Veerle E. I. Huyghe, Arpine Hovasapian, Johnny R. J. Fontaine

**Affiliations:** Department of Work, Organization and Society, Faculty of Psychology and Educational Sciences, Ghent University, Ghent, Belgium

**Keywords:** method effects, acquiescence, scenario specificity, confirmatory factor analysis, modeling, emotional intelligence, emotional understanding

## Abstract

The internal structure of ability emotional intelligence (EI) tests at item level has been hardly studied, and if studied often the predicted structure did not show. In the present study, an *a priori* model for responses to EI ability items using Likert response scales with a Situational Judgement Test (SJT) format is investigated with confirmatory factor analysis. The model consists of (1) a target EI ability factor, (2) an acquiescence factor, which is a method factor induced by the Likert response scales, and (3) design-based error covariances, which are induced by the SJT format. It is investigated whether this *a priori* model can account for the observed associations between the raw item responses of the Components of Emotion Understanding Test-24 (CEUT-24). The CEUT-24 is a new test developed to assess emotion understanding, a key aspect of the EI ability construct, based on the componential emotion framework. The sample consisted of 1184 participants (15–22 years old) from four European countries (United Kingdom, Belgium, Germany, and Spain) speaking four different languages (English, Dutch, German and Spanish). Findings showed that the *a priori* model fitted the data well in all four languages. Furthermore, measurement invariance testing gave evidence for a well-fitting configural, metric, and partial scalar invariance model. The conclusion is that within a regular CFA framework using raw observed items responses, method factors (acquiescence response style and scenario induced variance) can be disentangled from the targeted EI ability factor.

## Introduction

Salovey and Mayer (1990, p. 185) introduced the concept of Emotional Intelligence (EI) as “a set of skills hypothesized to contribute to the accurate appraisal and expression of emotion in oneself and in others, the effective regulation of emotion in self and others, and the use of feelings to motivate, plan, and achieve in one’s life”. Using maximum performance assessment instruments empirical evidence has been found for its validity, showing substantial intercorrelations between EI subtests (e.g., Mayer et al., 2003; MacCann et al., 2014), small to moderate correlations with personality, and moderate to large correlations with intelligence (e.g., Lopes et al., 2003; Brackett et al., 2004; Mayer et al., 2004; MacCann et al., 2014).

However, one important issue has largely remained understudied: the internal structure of EI (sub) tests at item level (cfr., Maul, 2012a). The few studies that looked at the internal structure at item level often did not confirm the expectations or tended to find rather difficult to interpret structures (e.g., Follesdal and Hagtvet, 2009; Ferguson and Austin, 2011).

The difficult to interpret internal structures at item level can be linked to the practice of substituting raw responses to ability EI items by (a score derived from) the proportion of participants within a community sample or an expert sample that selected the respective responses (e.g., Mayer et al., 2003). This practice is based on two assumptions: (1) the consensus in a community or an expert sample contains valid information about correctness in the EI domain and (2) the transformation of individual raw responses into scores based on this community and/or expert information offers an unbiased score for the correctness of these responses.

Although still highly debated (cfr., Maul, 2012b), there is some support for the first assumption (e.g., community and expert samples agree largely about the correctness in the EI domain; Mayer et al., 2003). Regarding the second assumption, however, Legree et al. (2014) showed that for EI (sub)tests using Likert response scales (Supplementary Material Point 1), this practice introduces bias. These authors proposed to look at the profile of responses across items in an EI subtest. They identified three important properties of this profile: shape (i.e., pattern of scores across items), elevation (i.e., mean score across items), and scatter (i.e., variance of scores across items). Based on the theoretical analysis of what the MSCEIT pretends to measure (Mayer et al., 2002, 2003), Legree et al. (2014) claimed that only shape contains valid information about EI, while individual differences in elevation and scatter do not. These latter characteristics can be affected by other personality characteristics (such as response tendencies). The individual item score (and thus also the score into which it is transformed) confounds information about shape, elevation, and scatter, and therefore does not offer unbiased information about a person’s EI ability. To avoid this confounding, Legree et al. (2014) proposed to use Profile Similarity Metrics (PSM; Cronbach and Gleser, 1953). The similarity between the correct and the observed shape (or profile) of raw responses across items (e.g., by a computing a Pearson correlation) is not affected by score elevation and scatter, and thus should give unbiased information about an EI ability. While Legree et al. (2014) demonstrated that the profile similarities indeed contain valid information about EI abilities, a major limitation is that a profile similarity is a Gestalt measure. They give no information about the dimensionality of an EI (sub)test, as profiles can be meaningfully computed for both uni- and multidimensional tests. Moreover, they do not allow to investigate the psychometric quality of individual items, as it is only the profile across items that is deemed relevant.

Based on the work of Legree et al. (2014), who have demonstrated that raw item scores contain valid information about EI abilities (although not exclusively), a simple theoretical model for the constructs that determine associations between the raw Likert-type item scores is tested with confirmatory factor analysis (CFA) in the current study. This approach allows to test the dimensionality of the internal structure at item level and to evaluate the psychometric properties of individual items.

This *a priori* model identifies three constructs to account for reactions to EI items: (1) EI ability, (2) acquiescence, and (3) scenario specificity. The first construct, the target construct, is the EI ability. It can be predicted that the higher a participant’s EI ability, the more likely the participant will score correct items as correct and incorrect items as incorrect on Likert-type response scales. This will result in positive correlations between correct items, positive correlations between incorrect items, and negative correlations between correct and incorrect items. In a factor analytic model, the EI construct should generate a bipolar factor on which correct items load positively, and incorrect items load negatively.

The second construct is acquiescence, a response set which was already defined by Cronbach (1946, p. 479) as “tendency to use Yes or True”. It is known that when a Likert response scale is used, participants differ in their tendency to more or less “agree” with the items independent of the content of the items (e.g., Park and Wu, 2019). The more participants use the response scale to the higher end of the scale, irrespective of the content of the items, the higher their average score across items (cfr., elevation, Legree et al., 2014). In case of perfectly balanced instruments using conceptually opposite items (e.g., Weijters et al., 2010), the impact of acquiescence will cancel out in the average item score. However, in unbalanced instruments (i.e., more correct than incorrect items), the average item score will be determined by both EI ability and acquiescence. Interindividual differences in acquiescence will lead to positive correlations among all items and can be represented in CFA by a unipolar factor on which all items have the same loading (e.g., Billiet and McClendon, 2000; Weijters et al., 2010).

A third source of variance is scenario specificity. EI (sub)tests typically work with a situational judgement test (SJT) format in which items are nested in item stems (e.g., emotion-eliciting scenarios). In a factor analytic model, this design feature leads to the violation of local independence (e.g., Cole et al., 2007) and can be modeled by error covariances between items that share the same item stem.

Thus, in order to adequately represent the processes that account for the responses to EI items using a Likert response scale, a factor analytic model is proposed that consists of a bipolar EI ability factor, a unipolar acquiescence factor, and error covariances between items sharing the same item stem.

Using exploratory methods (Principal Component Analyses), Fontaine et al. (2021, submitted manuscript)^1^ identified a bipolar EI component and a unipolar acquiescence component in existing instruments, like the MSCEIT. The current study takes the next step: Using CFA, it is investigated whether the *a priori* model can actually account for the observed relationships between EI items that use a Likert response scale. Here the *a priori* model is tested with the Components of Emotional Understanding Test-24 (CEUT-24), which is a shortened 24 item version of the Components of Emotional Understanding Test (Sekwena and Fontaine, 2018). This instrument offers an appropriate case for testing the hypotheses about the constructs that determine raw scores to EI ability items because: (1) emotional understanding (EU) is a core aspect of EI (Salovey and Mayer, 1990), (2) the CEUT-24 is a perfectly balanced instrument with as many incorrect as correct items allowing identification of acquiescence independently from EI, (3) it has been developed based on extensive cross-cultural research, and (4) is embedded in a strong theoretical emotion framework.

The CEUT is based on the componential emotion approach (CEA), which offers a comprehensive theoretical framework about the nature of emotions (Fontaine et al., 2007). According to the CEA, an emotion is conceptualized as an interplay between five components (appraisals, action tendencies, bodily reactions, expressions, and subjective feelings) that are elicited by goal-relevant events (e.g., Scherer, 2009). The CEA is empirically supported by psycholinguistic research in 27 countries, 23 languages, and 34 samples (Fontaine et al., 2007, 2013). For 24 commonly used emotion terms, participants indicated the likelihood that 142 features representing each of the five components could be inferred when the emotion terms are used in their respective languages. This research showed that across languages and countries emotion terms refer consistently to variations in all five emotion components and that profiles of likely and unlikely features are encoded in languages around the world. Based on the CEA and the empirical cross-cultural psycholinguistic research, Fontaine (2016) proposed to redefine the concept of emotional understanding (EU)—which is a key part of the EI construct (e.g., Mayer et al., 2002)—as “understanding the likely appraisals, action tendencies, bodily reactions, expressions, and feelings in response to goal-relevant situations (p. 333)”.

The CEUT is a situational judgement test (SJT) in which participants are asked to imagine characters in 10 specific emotion-eliciting situations and to rate the likelihood that the main character in each situation would display a number of emotional reactions representing each of the five components (Sekwena and Fontaine, 2018). The scenarios were constructed based on extensive qualitative research among Black and White participants in South Africa. These scenarios represent a large variability of emotions (joy, anger, sadness, fear, surprise, compassion, pride, guilt, shame and love/friendship). Five emotion terms and five emotion features per emotion component that varied in terms of likelihood (ranging from very likely to very unlikely) were selected for each scenario based on the extensive cross-cultural psycholinguistic research (Fontaine et al., 2013). Validation research with the CEUT, using both proportion scoring and profile similarities per emotion component, revealed a unidimensional EU factor and confirmed by and large the expected relationships with cognitive, personality, and wellbeing measures (Sekwena and Fontaine, 2018).

The CEUT-24 is a shortened version with only six emotion-eliciting scenarios and four emotion features per scenario. The balanced nature of the original design was preserved: across the six scenarios each emotion component is represented by four features and half of the features are unlikely (incorrect) while half of the features are likely (correct).

Based on the theoretical expectations about the constructs determining item responses in EI ability tests with Likert response scales, it is thus predicted that a bipolar EU ability factor (likely emotional reactions load positively and unlikely emotional reactions load negatively), a unipolar acquiescence factor (on which all items have the same loading), and error covariances between items sharing the same emotion scenario will adequately represent the internal structure of the CEUT-24.

This study forms a part of a larger validation study in which shortened versions of assessment instruments were investigated for usage in four European countries (UK, Germany, Spain, and Belgium) and in four different languages (English, German, Spanish, and Dutch) within the Horizon2020 project “Assessing and Enhancing Emotional Competence for Well-Being (ECoWeB) in the Young: A principled, evidence-based, mobile-health approach to prevent mental disorders and promote mental well-being” (Newbold et al., 2020; Supplementary Material Point 2). Therefore also measurement invariance (MI) is investigated. Since both the scenarios and the emotion reactions were constructed on the basis of extensive cross-cultural research (Fontaine et al., 2013), configural MI is expected for these four European languages. This is the minimal level of MI that is required to validly use the instrument within each of the four countries (e.g., Schmitt and Kuljanin, 2008). Whether higher levels of metric (i.e., identical factor loadings), scalar (i.e., identical intercepts), and strict (i.e., identical error variances and covariances between items) MI hold, will be exploratively investigated.

## Materials and Methods

The current manuscript only focuses on the CEUT-24 data, within the validation study of the ECoWeB project.

### Participants

This study included 1184 participants (*n*_Belgium_ = 525; *n*_UK_ = 237; *n*_Germany_ = 209; *n*_Spain_ = 213), of which 12 participants did not fill in their gender. The gender distributions were more or less equally split across female and male (percentage female: Belgium: 49.8%; UK: 53.4%; Germany: 55.1%; Spain: 50.2%). In Belgium, the age range was between 15 and 22 (mean age = 17.64), while in the other 3 countries the age range was between 16 and 22 years old (mean ages: UK = 19.34, Germany = 19.22, Spain = 19.31) (see also Supplementary Material Point 3).

### Procedures

In Belgium, the research team collaborated with students from the Psychology course “Assessment Theory.” These students each recruited one participant who was between 15 and 22 years old to participate in an online psychological assessment. Informed consent was obtained both on paper (for minors we also collected parental informed consent forms) and digitally. In the UK, Spain, and Germany, Qualtrics Research Services was hired to recruit (at least) two hundred 16–22-year-old participants. Two hundred participants were set as minimal target as it is often considered as a reasonable sample size to provide sufficient statistical power in structural equation modeling (e.g., Hoe, 2008; Kyriazos, 2018). Minors were invited or recruited through their parents. The survey started with a general introduction and a digital informed consent.

### Ethical Considerations

The Ethical Committee of the Faculty of Psychology and Educational Sciences of Ghent University has confirmed that the research was conducted according to the ethical rules presented in its General Ethical Protocol (see waiver 2020/93).

### Instruments

#### Components of Emotional Understanding Test-24 Item Version

The CEUT-24 is a short form of the original CEUT (Sekwena and Fontaine, 2018), and consists of six emotion-eliciting scenario’s (i.e., the item stems) and four possible emotional reactions of the main character in the scenario (i.e., the items). The items have to be rated on the following rating scale: (1) very unlikely, (2) unlikely, (3) neither likely nor unlikely, (4) likely, and (5) very likely (i.e., numbers 1–5 being the raw item scores, with scores 1 and 2 being correct when an item is unlikely and scores 4 and 5 being correct when an item is likely).

The International Test Commission guidelines regarding adaptation and translation (Hernandez et al., 2020) were followed (Supplementary Material Point 4). The English version and an already existing Dutch version of the CEUT made under the supervision of Sekwena and Fontaine were taken as the source versions for edits in the context of the ECoWeB project. This was done by the Belgian ECoWeB team, consisting of both Dutch and English native speaking emotion researchers. The English CEUT-24 was then translated by the Spanish and German ECoWeB collaborators using back translations and committee discussions.

The reliability, Mac Donald’s omega, computed with the SPSS macro from Hayes and Coutts (2020) was 0.83, 0.94, 0.89, and 0.93 in Belgium, UK, Germany, and Spain, respectively.

### Data Analysis

For CFA analyses, MPlus (Muthén and Muthén, 2011) was used. Since the items show a skewed distribution, most pronounced in the Belgian sample, we used MLMV estimation (Maydeu-Olivares, 2017; Supplementary Material Point 5).

For evaluating the fit indices, the criteria suggested by Schweizer (2010) were used. The normed chi-square should be below 2 to indicate good model fit and below 3 to indicate acceptable model fit. The root mean square error of approximation should be less than 0.05 for good model fit and less than 0.08 for acceptable model fit. The Comparative Fit Index (CFI) needs to have a value between 0.95 and 1.00 for good model fit and between 0.90 and 0.95 for acceptable model fit. Finally, the standardized root mean square residual (SRMR) should be below 0.10.

## Results

**Figure 1:**
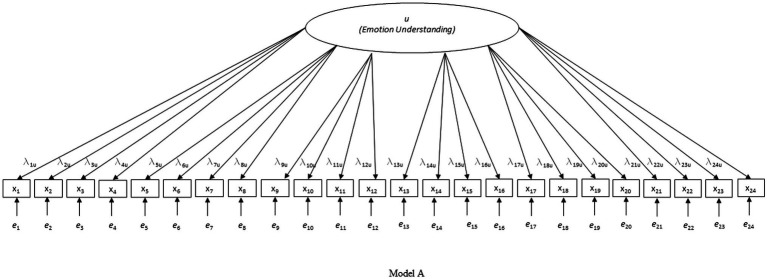
Investigated model A.

**Figure 2:**
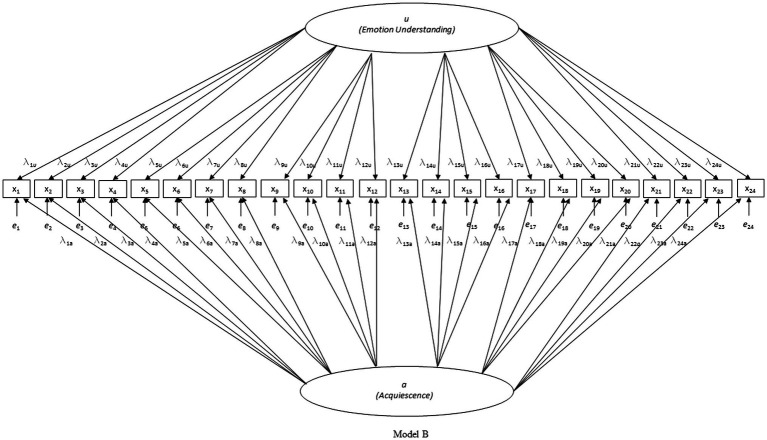
Investigated model B.

**Figure 3:**
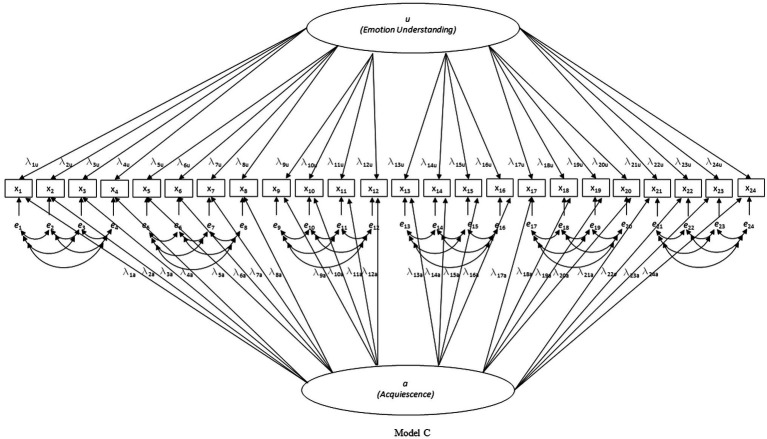
Investigated model C (hypothesized model).

First, three nested models were tested for each country separately (see Figures 1–3): model A with only the EU factor, model B with the acquiescence factor added to model A, and model C with the scenario-specific error covariances added to model B (hypothesized model). Only model C had acceptable to good fit on all criteria in all four countries (except for the CFI in the Belgian sample which was slightly below 0.90; see Table 1). Due to the pronounced skewedness and kurtosis in the Belgian sample, model C was also estimated with WLSMV instead of MLMV. With WLSMV model C showed an even slightly better fit: *χ*^2^ = 392.283, df = 215, *χ*^2^/df = 1.825; RMSEA = 0.040; RMSEA 90% CI = 0.033–0.046; CFI = 0.957; SRMR = 0.048.

**Table 1:** Goodness-of-fit statistics for the measurement models.

		Fit indices
		χ^2^	df	χ^2^/df	RMSEA	RMSEA CI^*^	CFI	SRMR
Separate groups	Model							
*Belgium*	*A*	659.546	252	2.617	0.056	0.050–0.061	0.590	0.073
	*B*	627.715	251	2.501	0.053	0.048–0.059	0.621	0.070
	*C*	337.178	215	1.568	0.033	0.026–0.039	0.877	0.045
*Germany*	*A*	565.539	252	2.244	0.077	0.069–0.086	0.599	0.110
	*B*	432.983	251	1.725	0.059	0.049–0.068	0.767	0.081
	*C*	252.649	215	1.175	0.029	0.007–0.043	0.952	0.049
*Spain*	*A*	586.877	252	2.329	0.079	0.071–0.087	0.633	0.105
	*B*	450.661	251	1.795	0.061	0.052–0.070	0.781	0.076
	*C*	253.304	215	1.178	0.029	0.008–0.042	0.958	0.043
*UK*	*A*	493.394	252	1.958	0.064	0.055–0.072	0.735	0.082
	*B*	410.761	251	1.636	0.052	0.043–0.061	0.825	0.063
	*C*	255.496	215	1.188	0.028	0.009–0.041	0.956	0.046
Across groups	Model C							
*Configural*		1081.270	860	1.257	0.029	0.024–0.035	0.938	0.045
*Metric*		1195.595	929	1.287	0.031	0.026–0.036	0.925	0.091
*Scalar*		1473.420	995	1.481	0.040	0.036–0.045	0.866	0.130
*Partial Scalar*		1245.805	962	1.295	0.032	0.026–0.036	0.921	0.092
*Strict*		2061.629	1175	1.755	0.050	0.047–0.054	0.752	0.128

*Model A is the model with only the EU factor, Model B has both the EU and the acquiescence factor, and Model C is Model B with additionally error covariances between items from the same item stem. Partial Scalar is with 11 intercepts freely estimated (a4 b3 c1 c2 c3 c4 d2 d3 d4 e1 f3)*.

* *RMSEA CI refers to the 90% confidence interval for the RMSEA*.

**Figure 4:**
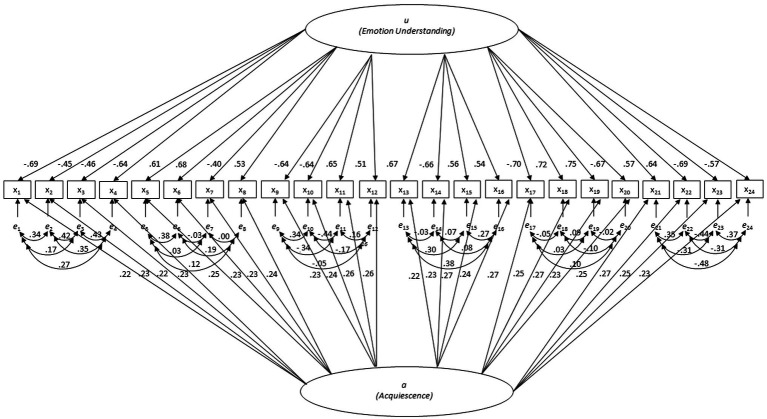
Metric measurement invariance model for UK with standardized factor loadings.

Subsequently, the configural, metric, scalar, and strict MI models were tested across the four countries using MLMV (see Table 1). In line with the analyses per sample and with our expectations, all fit criteria showed acceptable to good fit for configural invariance. When the factor loadings were additionally restricted to be the same in all samples (metric MI), all fit criteria still showed acceptable to good fit. However, when the intercepts were additionally restricted, both the CFI and the SRMR showed lack of model fit. The fit further worsened for the strict MI model with invariant error variances and covariances. The metric MI model for UK is depicted in Figure 4 (standardized factor loadings) and Table 2 (unstandardized factor loadings).

**Table 2:** Unstandardized factor loadings of the metric invariance model in the UK sample.

	*EU factor*	*Acquiescence factor*
A1	−1.27	1.00
A2	−0.80	1.00
A3	−0.86	1.00
A4	−1.14	1.00
B1	1.00	1.00
B2	1.17	1.00
B3	−0.72	1.00
B4	0.91	1.00
C1	−1.11	1.00
C2	−1.09	1.00
C3	1.02	1.00
C4	0.79	1.00
D1	1.22	1.00
D2	−1.16	1.00
D3	0.84	1.00
D4	0.91	1.00
E1	−1.04	1.00
E2	1.17	1.00
E3	1.14	1.00
E4	−1.19	1.00
F1	0.93	1.00
F2	0.98	1.00
F3	−1.13	1.00
F4	−1.02	1.00

*A = situation 1; B= situation 2, C= situation 3, D= situation 4, E = situation 5, and F = situation 6*.

Finally, it was investigated whether partial scalar MI could be identified based on the modification indices. When 11 of the 24 intercepts were freed, the model fitted about as well as the metric invariance model (see Table 1). The partial scalar MI allows to compare latent means between countries. The latent factor mean on the EU factor of the Belgian sample was significantly higher than of the other three samples, while these other three samples did not differ significantly from each other (see Supplementary Material Point 6).

## Discussion

### Modeling Likert Scale Responses to EI Ability Items

The predicted bipolar EU ability factor clearly emerged in all four languages. As hypothesized, the higher people score on the EU ability factor the higher they rate likely emotional reactions and the lower they rate unlikely emotional reactions on the Likert response scale. The fit measures indicated that the EU factor on its own was insufficient to account for the observed covariances between the EU ability items. The two design-based method factors—acquiescence because of the Likert response scale and error covariances because of the nesting of emotion reactions in specific scenarios—had to be included both in all four languages to arrive at a well-fitting model.

Because the CEUT-24 is the first balanced EU ability instrument, the impact of acquiescence is independent from EU ability (Weijters et al., 2010). When a simple sum score across all items (after reverse scoring unlikely emotional reactions) is computed as a proxy for EU ability, the effect of acquiescence is canceled out. The fact that the design-based covariances had to be included for a well-fitting model, means that participants also systematically varied in how they interpreted the individual scenarios over and above their general EU ability. This scenario-specific sensitivity can be accounted for by very different psychological constructs, such as personality traits, preferences, and previous personal experiences with comparable situations. These error covariances could introduce some bias when the sum score is used as a proxy for the EU ability. Still, since these error covariances are limited to the items that belong to the same scenario, it can be expected that most of their impact will cancel out across scenarios. With an omega over 0.80 in two samples and over 0.90 in the two other samples, the CEUT-24 shows satisfactory reliability for a short scale of only 24 items.

The current results go beyond the earlier findings of Fontaine et al. (2021, submitted manuscript; see footnote 1) who identified a bipolar EI component and a unipolar acquiescence component using exploratory methods. It is shown that with a well-designed balanced instrument, a bipolar EI ability factor, a unipolar acquiescence factor, and scenario-specific error covariances, cannot only be identified, but also account for the observed associations between the items. Demonstrating that there is indeed only one EU ability factor that accounts for the responses toward very different emotional reactions in very different situations, as predicted by the EI construct, contributes to the validity of the CEUT-24. The current results further clearly indicate that there is no need to transform the raw item scores to proportions or to restrict oneself to profile similarities (Legree et al., 2014). The raw item scores contain all information that is needed to model the predicted content and method factors.

### Measurement Invariance

As expected, the internal structure replicated well in each of the four groups. In addition, the metric measurement invariance (MI) model also showed good fit. This means that latent changes on the CEUT-24, for instance due to an intervention, can be interpreted in the same way in each of the four languages. The lack of full scalar and of strict MI, however, points to some impact of language and possibly cultural factors on the interpretation of the items. Still, as the partial scalar MI holds, it is possible to directly compare the groups on the latent variables. In the current study, the latent differences between the four groups probably point to sampling factors, rather than cultural or linguistic factors, as there was only a significant difference between the Belgian sample, which was collected by students, and the three other samples, which were collected by the Qualtrics service.

### Limitations

A limitation of the current study is that the assessment of the possible impact of skewedness and kurtosis could not be properly investigated in the UK, German, and Spanish samples as their sample sizes were too small. The Belgian sample, where skewedness and kurtosis were most pronounced, was sufficiently large to work with the WLSMV estimator (cfr., Li, 2016). This showed the predicted model was equally well supported. It would be interesting in the future to study larger samples and to further investigate MI using the WLSMV estimator.

It might be noted that the observation of a higher average ability in the Belgian sample and the more pronounced skewedness and kurtosis in this sample probably points to an inherent relationship. The higher the average EI ability in a sample, the more participants will either choose the highest (for correct item) or the lowest (for incorrect item) rating and thus the more the item distributions will be positively or negatively skewed and show more kurtosis. This means that when high EI ability groups are studied, larger samples are required.

### Future Directions

Since the results show that the proposed model accounts for the associations between the CEUT-24 raw item responses, an important future perspective is that the psychometric properties of individual EI ability items and how they contribute to the overall assessment of the EI ability can be investigated. Moreover, since EI ability can now be differentiated from acquiescence, the impact of both on the nomological network can be disentangled in the future. Additionally, further research should not be limited to the CEUT-24, but be extended to other existing EI (sub)tests. It can be investigated whether the same model holds for existing EI ability instruments with an SJT format using Likert response scales. Furthermore, it will be possible to study the internal structure of the whole EI domain and compare different (hierarchical) factor models [cfr., MacCann et al. (2014) versus Mayer et al. (2002)] starting from the lowest level of the item scores.

## Conclusion

The present study offers a simple, robust model for the factors that account for raw responses to EI ability items in the CEUT-24. By demonstrating that this model fits well in four different languages, it supports the claim that only one EI ability (emotion understanding) is assessed, which can be disentangled from method factors.

## Data Availability Statement

The datasets presented in this article are not readily available because the ECoWeB project is still ongoing. Requests to access the datasets should be directed to johnny.fontaine@ugent.be. The data will be stored in a repository after the end of the ECoWeB project (the data will be made available without undue reservation).

## Ethics Statement

The Ethical Committee of the Faculty of Psychology and Educational Sciences of Ghent University has confirmed that the research was conducted according to the ethical rules presented in its General Ethical Protocol (see waiver 2020/93). The participants provided their informed consent, either digitally or written, to participate in this study.

## Author Contributions

VH was the lead author on the manuscript and coordinated the recruitment and collaboration with the Psychology course Assessment Theory (JF is professor of this course) for the Belgian sample. JF analyzed the data and contributed to preparing the grant proposal for the ECoWeB project. AH coordinated the recruitment and collaboration with Qualtrics Research Services for the German, Spanish and UK sample. AH and VH set up the logistics and online survey for the data collection. All authors contributed to the interpretation of the data, the writing of the manuscript and approved the final version.

## Funding

This work was supported by the Horizon 2020 ECoWeB project (grant agreement no. 754657). The study sponsor and funder have no influence on the conduct, delivery or reporting of the research.

## Conflict of Interest

The authors declare that the research was conducted in the absence of any commercial or financial relationships that could be construed as a potential conflict of interest.

## Publisher’s Note

All claims expressed in this article are solely those of the authors and do not necessarily represent those of their affiliated organizations, or those of the publisher, the editors and the reviewers. Any product that may be evaluated in this article, or claim that may be made by its manufacturer, is not guaranteed or endorsed by the publisher.

## Footnotes

^1^Fontaine, J. R. J., Sekwena, E., Veirman, E., Schlegel, K., MacCann, C., Roberts, R. D., and Scherer, K. R. (2021). *Assessing emotional intelligence abilities, acquiescent and extreme responding in situational judgement tests using Principal Component* Metrics [Manuscript submitted for publication].
