# Supplementary material for: The Scoring Challenge of Emotional Intelligence Ability Tests: A Confirmatory Factor Analysis Approach to Model Substantive and Method Effects Using Raw Item Scores
Source: Front Psychol. 2022 Mar 10;13:812525. doi: 10.3389/fpsyg.2022.812525 (PMC8960726; doi:10.3389/fpsyg.2022.812525)
Supplement: Supplementary file 1 [file Data_Sheet_1.pdf]

Supplementary Material

1 Likert scales

A Likert response scale is a very common response format in the EI domain. For instance, the Mayer-Salovey-Caruso Emotional Intelligence Test (MSCEIT; Mayer et al., 2002), which is considered the golden standard for assessing EI (O' Connor et al., 2019), uses a Likert scale response format for six of the eight subtests.

2 ECoWeB project and Ghent University team tasks

This project has received funding from the European Union's Horizon 2020 research and innovation program under grant agreement No 754657. In the first stage of this large European project, an app was developed to train emotional competence (MyMoodCoach app) and an assessment battery was prepared and programmed in Qualtrics for baseline and follow-up assessments to capture emotional competence.

Within the larger European-wide ECoWeB project, the UGent team has the following objectives: 'Development and validation of digital instruments and interventions for the assessment and training of the ability to identify and understand emotion processes in other people and in oneself on the basis of informative cues (knowledge component of the Emotional Competence construct), suitable to be used by young people' (cited from the projects' Grant Agreement).

3 More details regarding section Participants

3.1 Information on data cleaning and exclusions

First, the four data files were screened and cleaned. The following exclusion criteria were used to clean the data sets:

- Tryout cases to check the survey were deleted.
- Data entries with a progress below 40% of the survey were deleted.
- When participants restarted the survey, only the most complete data entry was kept.
- Cases of participants below 18 years old who had a missing Informed Consent form (5 participants) were deleted (applied only to the Belgian dataset).
- Cases of participants linked to students with whom irregularities were observed (3 participants) were deleted (applied only to the Belgian dataset).

Next, the four separate data files were integrated and further steps were taken:

- Participants younger than 15 years old and older than 22 years old were excluded (7 participants).
- Participants with missing values for the CEUT-24 were excluded (1 participant).
- For each participant the frequency of each of the response categories across the 24 items was computed. Participants that used more than 19 times the same response option (out of 24

times), were considered to not have carefully taken the test and were removed (26 participants).

These steps resulted in a final dataset of 1184 participants that was used for the MPlus analyses.

3.2 Ethnic identification

In all four samples participants mainly choose 'White' (87.8%) when asked about ethnic identification. In Table 1, the proportions for the different ethnic identification categories per country are reported.

Table 1

Ethnic Identification Data of Participants in Belgium, the UK, Germany, and Spain

	Belgium	UK	Germany	Spain	Total
White	93.9%	74.3%	91.9%	84%	87.8%
Mixed	2.7%	5.5%	1.9%	9.9%	4.4%
Asian	0.4%	11.4%	5.3%	0.5%	3.5%
Black	0.4%	5.5%	0.0%	1.4%	1.5%
Arab	1.9%	0.8%	0.5%	1.9%	1.4%
Other	0.6%	1.3%	0.5%	1.4%	0.8%
No reply	0.2%	1.3%	0.0%	0.9%	0.5%

Note. 'Other' means 'Other ethnic group', 'No reply' means 'Prefer not to say'

4 Translation and adaptation information CEUT to CEUT-24

4.1 Dutch CEUT-24

The Dutch CEUT-24 is based on an existing Dutch version of the CEUT (the South African Situational Test of Emotional Understanding; SASTEU; Sekwena & Fontaine, 2018). This existing Dutch version was developed by translation-back translation process by the original authors. The current CEUT-24 Dutch version has been derived from this existing Dutch version with small adaptations for the ECoWeB context: e.g., the terms for the emotion components are consistent with words used in the ECoWeB project (e.g., event evaluation became event appraisal; the addition of bodily posture to the definition of expression). In addition, a few small changes were made to the wording of some items.

4.2 CEUT-24 in English[1]

The base of the English version is a combination of the existing original English version of the CEUT (Sekwena & Fontaine, 2018), adapted to match the Dutch version for the CEUT-24.

Understanding emotional experiences

Instructions:

This questionnaire assesses your understanding of emotional experiences. This test looks at your understanding of five basic aspects of the emotion process:

1. **Event appraisal**: refers to the way the person evaluates or interprets the event that causes the emotion.
2. **Bodily reaction**: refers to how the body reacts during the emotion.
3. **Expression:** refers to how the emotion is expressed to others in the face, the voice, or the bodily posture.
4. **Action tendency**: refers to the tendency to behave in certain ways during the emotion.
5. **Subjective feeling**: refers to the feeling that characterizes the emotion.

In this test six emotional events are described that persons have experienced in the past. For each event four possible emotional reactions are presented. Please indicate for each reaction how likely it is that the person would have this emotional reaction in this situation. Please use the following response scale:

1	2	3	4	5
Very unlikely	Unlikely	Neither likely, nor unlikely	Likely	Very likely

You may now start with the test.

EVENT 1

Oliver is invited to a friend's birthday party at the student residence. After arriving at the venue, and looking at the decorations, he realizes that the party is actually organized for him by his friends. How likely would the following reactions be for Oliver at that moment?

How likely is it that he has the following EMOTION?

Disappointment

[1] The CEUT-24 can be freely used for scientific research non-profit purposes, but the authors ask to refer to this article and notify them before usage. The Dutch, German and Spanish versions can be requested from the corresponding author.

How likely is it that he has the following SUBJECTIVE FEELING?

He feels tired

How likely is it that he has the following BODILY REACTION?

He feels cold

How likely is it that he has the following ACTION TENDENCY?

He wants to destroy whatever is close.

EVENT 2

Alex is the first one to be accepted at university in her family. Her family pooled all their resources together to finance her studies. Upon arriving at the university, she enjoyed the student life and did not work very hard. At the end of the first year, she fails most of her modules. How likely would the following reactions be for Alex at that moment?

How likely is it that she has the following EMOTION?

Guilt

How likely is it that she has the following SUBJECTIVE FEELING?

She feels bad.

How likely is it that she EXPRESSES her emotion in the following way?

She speaks in a firm voice.

How likely is it that she has the following ACTION TENDENCY?

She wants to make up for what she has done.

EVENT 3

Chris experienced the master's program as challenging. At the end of the academic year he receives his final results. He completed his master degree with high merits. How likely would each of the following reactions be for Chris at that moment?

How likely is it that he has the following EMOTION?

Disappointment

How likely is it that he has the following EVENT APPRAISAL?

He thinks: 'This is an unpleasant situation for me.'

How likely is it that he has the following SUBJECTIVE FEELING?

He feels positive.

How likely is it that he has the following BODILY REACTION?

He feels warm.

EVENT 4

Harry receives the results of the semester test. He finds out that some correct answers have been marked wrong. Harry then asks the lecturer to rectify this and change the marks, but the lecturer refuses. How likely would the following reactions be for Harry at that moment?

How likely is it that he has the following EVENT APPRAISAL?

He thinks: 'I am treated unjustly’

How likely is it that he has the following SUBJECTIVE FEELING?

He feels calm.

How likely is it that he EXPRESSES his emotion in the following way?

He frowns.

How likely is it that he has the following ACTION TENDENCY?

He wants to oppose.

EVENT 5

Sarah has a special relationship with her uncle. The uncle regards Sarah as his child, since he does not have children of his own. One morning while at the university, Sarah receives a call from home saying that her uncle has suddenly passed away. How likely would the following reactions be for Sarah at that moment?

How likely is it that she has the following EVENT APPRAISAL?

She thinks: 'The consequences of this situation are positive, desirable for me.'

How likely is it that she has the following BODILY REACTION?

She feels a lump in her throat.

How likely is it that she EXPRESSES her emotion in the following way?

She has tears in her eyes.

How likely is it that she has the following ACTION TENDENCY?

She wants the ongoing situation to last or be repeated.

EVENT 6

Tess was recently attacked during the evening when walking back home from the university library. A few days later she receives an invitation from a good friend to attend an important reunion organized near the place where the attack happened. How likely would the following reactions be for Tess at the moment when she receives that invitation?

How likely is it that she has the following EMOTION?

Fear

How likely is it that she has the following EVENT APPRAISAL?

She thinks: 'This situation is unpleasant for me.’

How likely is it that she has the following BODILY REACTION?

Her muscles are relaxing.

How likely is it that she EXPRESSES her emotion in the following way?

She smiles.

4.3 Information regarding the Spanish translation

Two researchers, holding a PhD in Psychology, with expertise in research on psychological assessment and psychological treatments performed the translation of the CEUT-24 from English into Spanish. Then a native English speaker editing professional with expertise in translations of psychological texts from English to Spanish and vice versa conducted a back translation of the Spanish version of the CEUT-24 to English (this professional was hired to conduct this work). Finally, the two researchers reviewed the back translation to identify discrepancies that were corrected if needed.

4.4 Information regarding the German translation

One psychology researcher and one psychology student did a translation-backtranslation process for the German translation. Another ECoWeB team member from the UK team checked the back-translated English.

5 Means, Variances, Skewedness, Kurtosis and Covariances across language groups

Table 2

Means and Variances

Items	*Mean*				*Variance*			
	Belgium	*UK*	*Germany*	*Spain*	*Belgium*	*UK*	*Germany*	*Spain*
**A1**	1.450	1.726	1.651	1.681	0.807	1.288	1.165	1.100
**A2**	1.891	2.241	2.038	1.897	0.687	1.280	0.955	0.956
**A3**	1.863	2.241	2.129	1.901	0.804	1.347	0.945	0.972
**A4**	1.290	1.793	1.641	1.577	0.400	1.269	1.043	0.929
**B1**	4.554	4.346	4.139	4.315	0.426	0.834	1.009	0.910
**B2**	4.383	4.262	4.120	4.268	0.522	0.944	1.435	1.069
**B3**	1.933	2.793	2.301	2.338	0.519	1.067	1.005	0.853
**B4**	4.255	4.110	4.086	4.183	0.442	1.009	0.825	0.966
**C1**	1.385	1.700	2.608	1.563	0.705	1.239	1.138	1.072
**C2**	1.459	1.692	2.555	1.469	0.820	1.125	1.405	0.850
**C3**	4.714	4.384	3.502	4.493	0.356	0.844	1.159	0.795
**C4**	4.110	3.987	3.191	4.385	0.521	0.806	0.987	0.922
**D1**	4.566	4.329	4.330	4.357	0.432	0.947	1.283	1.075
**D2**	1.878	2.030	1.775	1.577	0.469	0.940	0.892	0.817
**D3**	4.063	4.148	3.751	4.164	0.379	0.717	0.905	0.813
**D4**	4.257	4.253	3.947	4.484	0.484	0.932	1.428	0.607
**E1**	1.120	1.544	1.330	1.423	0.144	1.058	0.575	0.817
**E2**	4.707	4.308	4.431	4.507	0.303	0.897	0.819	0.879
**E3**	4.781	4.494	4.555	4.620	0.221	0.748	0.888	0.715
**E4**	1.190	1.599	1.416	1.366	0.337	1.109	0.817	0.767
**F1**	4.244	4.245	4.010	4.117	0.440	0.885	0.995	0.883
**F2**	4.133	4.127	3.861	3.977	0.477	0.786	1.124	0.877
**F3**	1.899	1.806	1.833	1.709	0.845	1.009	1.039	0.713
**F4**	1.829	1.958	1.943	1.953	0.637	1.137	1.193	1.125

Note. A= situation 1; B= situation 2, C= situation 3, D = situation 4, E = situation 5, and F = situation 6.

Table 3

Skewedness and Kurtosis in the Four Samples

	Skewedness				*Kurtosis*			
	Belgium	***UK***	***Germany***	***Spain***	***Belgium***	***UK***	***Germany***	***Spain***
Items								
**A1**	2.44	1.44	1.75	1.42	5.80	.84	2.33	.88
**A2**	.41	.52	.51	.87	-.93	-.70	-.59	-.16
**A3**	.91	.61	.43	.82	.45	-.51	-.59	-.20
**A4**	2.48	1.21	1.60	1.68	6.48	.23	1.73	2.00
**B1**	-1.95	-1.67	-1.33	-1.57	5.96	2.78	1.39	2.21
**B2**	-1.48	-1.51	-1.47	-1.75	3.60	1.99	1.13	2.70
**B3**	.74	.24	.77	.68	1.11	-.48	.31	.47
**B4**	-.77	-1.35	-1.17	-1.44	1.40	1.65	1.48	1.95
**C1**	2.46	1.44	.00	1.94	5.68	.85	-.83	2.86
**C2**	2.43	1.47	.29	2.07	5.77	1.20	-.93	3.49
**C3**	-2.70	-1.55	-.71	-1.97	9.33	1.89	-.23	3.48
**C4**	-.56	-.82	-.36	-1.88	.60	.69	.05	3.40
**D1**	-2.03	-1.74	-1.64	-1.79	6.45	2.77	1.51	2.46
**D2**	.87	1.00	1.31	1.62	2.33	.80	1.47	1.85
**D3**	-.38	-1.25	-.89	-1.10	1.19	2.18	.77	.91
**D4**	-1.18	-1.51	-1.06	-2.09	3.27	2.05	.12	5.85
**E1**	3.53	1.84	2.33	2.27	13.97	2.25	4.42	4.54
**E2**	-2.07	-1.27	-1.81	-2.16	5.03	.82	2.92	4.14
**E3**	-2.27	-1.92	-2.23	-2.45	5.86	3.45	4.27	5.59
**E4**	3.75	1.70	2.16	2.45	15.83	1.83	3.60	4.94
**F1**	-1.18	-1.60	-1.15	-1.29	4.27	2.69	.89	1.66
**F2**	-.94	-1.05	-1.07	-.88	2.48	.91	.69	.46
**F3**	1.23	1.27	1.23	1.20	1.42	1.02	.82	1.16
**F4**	.70	1.02	.93	.99	.03	.26	-.08	.28

Table 4

Covariances Belgium

	A1	A2	A3	A4	B1	B2	B3	B4	C1	C2	C3	C4	D1	D2	D3	D4	E1	E2	E3	E4	F1	F2	F3	F4
**A1**	.807																							
**A2**	.138	.687																						
**A3**	.168	.368	.804																					
**A4**	.169	.195	.188	.400																				
**B1**	-.064	-.075	-.052	-.075	.426																			
**B2**	-.069	-.090	-.081	-.111	.270	.522																		
**B3**	.087	.124	.092	.082	-.115	-.165	.519																	
**B4**	-.088	-.054	-.072	-.036	.137	.127	-.118	.442																
**C1**	.398	.028	.018	.108	-.046	-.052	.056	-.054	.705															
**C2**	.091	.122	.084	.159	-.091	-.136	.130	-.062	.300	.820														
**C3**	-.066	-.056	-.022	-.104	.078	.111	-.084	.044	-.227	-.235	.356													
**C4**	-.113	-.108	-.168	-.093	.057	.085	-.076	.073	-.126	-.157	.150	.521												
**D1**	-.071	-.062	-.065	-.069	.142	.157	-.136	.109	-.056	-.081	.084	.073	.432											
**D2**	.106	.105	.113	.052	-.058	-.073	.114	-.068	.066	.064	-.037	-.040	-.131	.469										
**D3**	-.049	-.060	-.049	-.009	.079	.086	-.072	.051	-.043	-.040	.050	.065	.075	-.053	.379									
**D4**	-.114	-.046	-.073	-.065	.056	.083	-.107	.072	-.068	-.036	.054	.111	.159	-.113	.081	.484								
**E1**	.087	.059	.051	.062	-.049	-.057	.046	-.034	.089	.082	-.072	-.034	-.056	.057	-.015	-.035	.144							
**E2**	-.091	-.078	-.069	-.058	.079	.088	-.079	.075	-.068	-.065	.069	.053	.113	-.076	.066	.098	-.075	.303						
**E3**	-.063	-.060	-.078	-.047	.093	.099	-.095	.067	-.045	-.052	.063	.058	.120	-.080	.039	.072	-.061	.130	.221					
**E4**	.099	.066	.100	.101	-.045	-.063	.076	-.047	.121	.080	-.087	-.048	-.100	.122	-.020	-.074	.070	-.057	-.065	.337				
**F1**	-.092	-.040	-.052	-.052	.092	.124	-.073	.086	-.050	-.045	.054	.061	.121	-.092	.084	.069	-.033	.108	.095	-.060	.440			
**F2**	-.066	-.066	-.094	-.050	.077	.128	-.033	.078	-.038	-.033	.072	.052	.098	-.071	.053	.063	-.043	.111	.084	-.056	.198	.477		
**F3**	.123	.138	.064	.105	-.133	-.118	.077	-.049	.085	.073	-.097	-.048	-.080	.131	-.077	-.069	.058	-.100	-.070	.057	-.156	-.148	.845	
**F4**	.096	.139	.087	.069	-.065	-.096	.112	-.021	.070	.079	-.087	-.080	-.073	.090	-.050	-.080	.036	-.083	-.079	.061	-.179	-.175	.166	.637

Table 5

Covariances UK

	A1	A2	A3	A4	B1	B2	B3	B4	C1	C2	C3	C4	D1	D2	D3	D4	E1	E2	E3	E4	F1	F2	F3	F4
**A1**	1.288																							
**A2**	0.796	1.280																						
**A3**	0.661	0.807	1.347																					
**A4**	0.842	0.801	0.851	1.269																				
**B1**	-0.462	-0.260	-0.206	-0.279	0.834																			
**B2**	-0.494	-0.282	-0.274	-0.381	0.559	0.944																		
**B3**	0.327	0.413	0.252	0.375	-0.076	-0.140	1.067																	
**B4**	-0.476	-0.330	-0.153	-0.306	0.384	0.486	-0.112	1.009																
**C1**	0.774	0.599	0.604	0.752	-0.293	-0.373	0.330	-0.351	1.239															
**C2**	0.637	0.517	0.542	0.717	-0.366	-0.316	0.333	-0.283	0.811	1.125														
**C3**	-0.523	-0.379	-0.383	-0.469	0.395	0.435	-0.157	0.279	-0.598	-0.629	0.844													
**C4**	-0.320	-0.305	-0.233	-0.247	0.304	0.349	-0.104	0.347	-0.291	-0.354	0.376	0.806												
**D1**	-0.412	-0.332	-0.324	-0.438	0.283	0.332	-0.084	0.293	-0.420	-0.477	0.393	0.266	0.947											
**D2**	0.460	0.427	0.390	0.487	-0.225	-0.303	0.179	-0.206	0.473	0.402	-0.378	-0.194	-0.330	0.940										
**D3**	-0.424	-0.318	-0.225	-0.328	0.215	0.350	-0.151	0.376	-0.293	-0.326	0.243	0.247	0.445	-0.220	0.717									
**D4**	-0.504	-0.462	-0.331	-0.462	0.301	0.318	-0.142	0.327	-0.405	-0.458	0.312	0.189	0.554	-0.265	0.397	0.932								
**E1**	0.706	0.574	0.586	0.737	-0.340	-0.425	0.328	-0.376	0.703	0.644	-0.500	-0.318	-0.428	0.705	-0.308	-0.349	1.058							
**E2**	-0.557	-0.399	-0.331	-0.413	0.370	0.438	-0.160	0.477	-0.444	-0.323	0.413	0.316	0.405	-0.317	0.372	0.306	-0.488	0.897						
**E3**	-0.557	-0.368	-0.330	-0.417	0.327	0.436	-0.143	0.300	-0.426	-0.447	0.448	0.264	0.454	-0.344	0.294	0.390	-0.459	0.502	0.748					
**E4**	0.603	0.476	0.476	0.584	-0.300	-0.342	0.301	-0.264	0.585	0.547	-0.386	-0.246	-0.332	0.480	-0.249	-0.249	0.691	-0.417	-0.380	1.109				
**F1**	-0.355	-0.270	-0.219	-0.295	0.341	0.404	-0.135	0.340	-0.201	-0.296	0.286	0.421	0.329	-0.210	0.238	0.322	-0.281	0.389	0.360	-0.193	0.885			
**F2**	-0.391	-0.220	-0.195	-0.273	0.353	0.380	-0.134	0.315	-0.333	-0.277	0.352	0.259	0.330	-0.282	0.243	0.335	-0.356	0.476	0.435	-0.236	0.509	0.786		
**F3**	0.550	0.490	0.494	0.576	-0.296	-0.295	0.399	-0.299	0.651	0.573	-0.377	-0.251	-0.354	0.474	-0.296	-0.381	0.658	-0.447	-0.415	0.547	-0.417	-0.494	1.009	
**F4**	0.575	0.466	0.453	0.514	-0.264	-0.301	0.346	-0.253	0.511	0.510	-0.304	-0.313	-0.328	0.377	-0.255	-0.344	0.546	-0.367	-0.359	0.574	-0.530	-0.433	0.709	1.137

Table 6

Covariances Germany

	A1	A2	A3	A4	B1	B2	B3	B4	C1	C2	C3	C4	D1	D2	D3	D4	E1	E2	E3	E4	F1	F2	F3	F4
**A1**	1.165																							
**A2**	0.291	0.955																						
**A3**	0.361	0.459	0.945																					
**A4**	0.626	0.406	0.362	1.043																				
**B1**	-0.129	0.062	-0.056	-0.204	1.009																			
**B2**	-0.441	0.000	-0.202	-0.421	0.625	1.435																		
**B3**	0.287	0.165	0.205	0.285	-0.061	-0.194	1.005																	
**B4**	-0.233	0.011	-0.107	-0.290	0.304	0.444	-0.160	0.825																
**C1**	0.160	0.082	0.142	0.208	-0.003	-0.025	0.046	0.034	1.138															
**C2**	0.280	0.165	0.287	0.218	-0.077	-0.172	0.139	0.120	0.749	1.405														
**C3**	-0.155	0.110	0.007	-0.164	0.213	0.428	0.117	0.134	-0.573	-0.642	1.159													
**C4**	0.096	0.151	0.186	0.093	0.227	0.058	0.205	-0.007	-0.145	-0.269	0.387	0.987												
**D1**	-0.392	-0.065	-0.263	-0.508	0.614	0.827	-0.233	0.484	0.015	-0.121	0.332	0.037	1.283											
**D2**	0.462	0.162	0.330	0.450	-0.208	-0.327	0.274	-0.253	0.122	0.177	-0.040	0.062	-0.447	0.892										
**D3**	-0.154	-0.077	-0.178	-0.199	0.307	0.341	0.003	0.342	0.099	-0.010	0.101	0.052	0.561	-0.233	0.905									
**D4**	-0.377	-0.223	-0.209	-0.430	0.390	0.494	-0.094	0.354	0.065	-0.033	0.146	0.077	0.711	-0.371	0.418	1.428								
**E1**	0.359	0.140	0.244	0.353	-0.218	-0.255	0.192	-0.167	0.125	0.152	0.040	0.147	-0.291	0.395	-0.186	-0.298	0.575							
**E2**	-0.304	-0.093	-0.156	-0.286	0.457	0.489	-0.149	0.379	-0.065	-0.153	0.214	0.133	0.561	-0.248	0.294	0.353	-0.180	0.819						
**E3**	-0.313	-0.045	-0.163	-0.413	0.445	0.575	-0.220	0.431	-0.021	-0.040	0.228	0.076	0.654	-0.378	0.320	0.498	-0.274	0.493	0.888					
**E4**	0.456	0.156	0.252	0.508	-0.307	-0.433	0.319	-0.256	0.245	0.243	-0.137	0.179	-0.472	0.429	-0.160	-0.299	0.489	-0.371	-0.432	0.817				
**F1**	-0.289	0.038	-0.159	-0.245	0.477	0.559	-0.070	0.325	0.013	-0.001	0.215	0.032	0.590	-0.261	0.419	0.441	-0.223	0.374	0.373	-0.258	0.995			
**F2**	-0.412	-0.066	-0.145	-0.275	0.488	0.576	-0.197	0.352	0.041	0.048	0.094	0.084	0.649	-0.294	0.410	0.476	-0.227	0.510	0.469	-0.287	0.638	1.124		
**F3**	0.497	0.169	0.323	0.442	-0.307	-0.482	0.290	-0.301	0.092	0.136	-0.040	0.161	-0.461	0.517	-0.214	-0.325	0.395	-0.435	-0.386	0.543	-0.391	-0.478	1.039	
**F4**	0.458	0.232	0.280	0.372	-0.236	-0.295	0.395	-0.287	0.064	0.070	0.125	0.260	-0.292	0.489	-0.201	-0.180	0.402	-0.396	-0.327	0.498	-0.454	-0.424	0.636	1.193

Table 7

Covariances Spain

	A1	A2	A3	A4	B1	B2	B3	B4	C1	C2	C3	C4	D1	D2	D3	D4	E1	E2	E3	E4	F1	F2	F3	F4
**A1**	1.100																							
**A2**	0.549	0.956																						
**A3**	0.508	0.610	0.972																					
**A4**	0.659	0.487	0.508	0.929																				
**B1**	-0.350	-0.188	-0.105	-0.341	0.910																			
**B2**	-0.422	-0.127	-0.110	-0.338	0.723	1.069																		
**B3**	0.343	0.213	0.292	0.373	-0.186	-0.184	0.853																	
**B4**	-0.200	-0.056	-0.113	-0.186	0.492	0.467	-0.137	0.966																
**C1**	0.508	0.312	0.314	0.468	-0.332	-0.367	0.331	-0.253	1.072															
**C2**	0.403	0.325	0.305	0.386	-0.246	-0.276	0.278	-0.302	0.613	0.850														
**C3**	-0.336	-0.142	-0.149	-0.205	0.422	0.469	-0.120	0.426	-0.339	-0.396	0.795													
**C4**	-0.225	-0.148	-0.108	-0.180	0.452	0.470	-0.097	0.474	-0.377	-0.340	0.599	0.922												
**D1**	-0.365	-0.151	-0.171	-0.291	0.456	0.515	-0.102	0.414	-0.342	-0.322	0.439	0.473	1.075											
**D2**	0.494	0.318	0.339	0.418	-0.360	-0.422	0.359	-0.326	0.440	0.522	-0.355	-0.340	-0.474	0.817										
**D3**	-0.347	-0.138	-0.209	-0.203	0.441	0.501	-0.173	0.392	-0.243	-0.265	0.440	0.416	0.514	-0.372	0.813									
**D4**	-0.193	-0.110	-0.126	-0.218	0.331	0.359	-0.084	0.334	-0.221	-0.213	0.325	0.410	0.438	-0.293	0.413	0.607								
**E1**	0.487	0.321	0.356	0.385	-0.339	-0.343	0.350	-0.312	0.508	0.511	-0.293	-0.266	-0.254	0.512	-0.257	-0.181	0.817							
**E2**	-0.402	-0.257	-0.213	-0.222	0.474	0.465	-0.106	0.428	-0.361	-0.346	0.525	0.551	0.519	-0.467	0.504	0.393	-0.388	0.879						
**E3**	-0.361	-0.255	-0.239	-0.325	0.397	0.430	-0.130	0.384	-0.438	-0.329	0.432	0.442	0.455	-0.400	0.372	0.344	-0.375	0.517	0.715					
**E4**	0.530	0.338	0.346	0.451	-0.322	-0.375	0.313	-0.302	0.512	0.481	-0.312	-0.329	-0.370	0.530	-0.271	-0.205	0.592	-0.406	-0.457	0.767				
**F1**	-0.225	-0.077	-0.073	-0.204	0.254	0.293	-0.190	0.119	-0.198	-0.130	0.280	0.302	0.320	-0.246	0.291	0.244	-0.148	0.433	0.364	-0.306	0.883			
**F2**	-0.238	-0.059	-0.073	-0.193	0.289	0.363	-0.166	0.178	-0.264	-0.177	0.312	0.347	0.351	-0.249	0.309	0.321	-0.173	0.355	0.348	-0.250	0.604	0.877		
**F3**	0.353	0.214	0.206	0.281	-0.232	-0.331	0.281	-0.139	0.352	0.320	-0.298	-0.235	-0.197	0.370	-0.224	-0.188	0.339	-0.284	-0.280	0.365	-0.426	-0.425	0.713	
**F4**	0.257	0.159	0.225	0.309	-0.267	-0.293	0.316	-0.151	0.332	0.271	-0.259	-0.137	-0.251	0.351	-0.157	-0.113	0.231	-0.178	-0.234	0.336	-0.459	-0.419	0.484	1.125

6 Pairwise comparisons latent means in the four groups

Regarding the significant differences: pairwise comparison between Belgium (reference group) and UK results in *m*= -0.249, *z*= -5.688 ($p < .001$), between Belgium and Germany in m= -0.216 ,*z*= -4.984 ($p < .001$), and between Belgium and Spain in m= -0.151, *z*= -3.531 ($p < .001$). The other three were non-significant (UK (reference group) compared to Germany results in *z*=0.589 ($p > .05$), UK compared to Spain results in *z*=1.699 ($p > .05$), and Germany compared to Spain results in *z*=1.130 ($p > .05$)).

7 Reference list

Mayer, J. D., Salovey, P., & Caruso, D. R. (2002). *Mayer-Salovey-Caruso Emotional Intelligence Test (MSCEIT). User's Manual.* Toronto, Canada: Multi-Health Systems.

O'Connor, P. J., Hill, A., Kaya, M., & Martin, B. (2019). The Measurement of Emotional Intelligence: A Critical Review of the Literature and Recommendations for Researchers and Practitioners. *Frontiers in Psychology*, *10*. https://doi.org/ARTN111610.3389/fpsyg.2019.01116

Sekwena, E. K., & Fontaine, J. R. J. (2018). Redefining and assessing emotional understanding based on the componential emotion approach. *South African Journal of Psychology*, *48*(2), 243-254. https://doi.org/10.1177/0081246317714681
